# Neuro-developmental outcome in sagittal synostosis; Analysis of 488 children in the context of the existing literature

**DOI:** 10.1007/s00381-026-07292-y

**Published:** 2026-05-04

**Authors:** May Brami, Sapir Sadon, Jonathan Roth, Jehuda Soleman, David Leshem, Shlomi Constantini

**Affiliations:** 1 Department of Pediatric Neurosurgery, The Pediatric Brain Center, Dana Children’s Hospital, Tel-Aviv Medical Center, Tel-Aviv University, Tel-Aviv, Israel; 2 Department of Pediatric Neurosurgery, University Children’s Hospital of Basel, Basel, Switzerland; 3 Faculty of Medicine University of Basel, Basel, Switzerland; 4 Department of Plastic and Reconstructive Surgery, Tel-Aviv Medical Center, Tel-Aviv University, Tel-Aviv, Israel; 5 The Pediatric Brain Center, Director: Gilbert Israeli International Neurofibromatosis Center, Dana Children’s Hospital, Tel Aviv Medical Center, 6, Weizman, 6423906 Tel-Aviv, Israel

**Keywords:** Craniosynostosis, Sagittal synostosis, Neurodevelopmental, Cognitive outcome, Neurocognitive outcome, Long-term follow-up

## Abstract

**Purpose:**

The neurodevelopmental impact of sagittal synostosis (SS) remains controversial, with conflicting evidence regarding long-term cognitive and functional outcome. This report reviews the literature on neurodevelopmental outcome in SS and presents our experience with 488 children who underwent open, wide, strip craniectomy and were evaluated for long-term functional outcome.

**Methods:**

A narrative literature review was conducted using PubMed and Google Scholar to identify studies reporting neurodevelopmental, cognitive, or functional outcomes in children with SS. In parallel, a retrospective cohort included children who underwent early, wide, open strip craniectomy for isolated SS between 1999 and 2022, with a minimum follow-up of two years. Functional outcomes were assessed using structured parental telephone questionnaires.

**Results:**

Twenty-two original research articles and six review studies met inclusion criteria, showing heterogeneous methodologies but generally normal long-term neurodevelopmental outcome with occasional mild, domain-specific vulnerabilities. In our cohort, 488 children (397 males) had a mean follow-up of 9.4 ± 6.3 years (range 2–25). The majority demonstrated normal *functional* development. Educational support was rarely required, and no neurodevelopmental or functional limitations related to surgery were identified in adulthood. Among 386 children of school age, mild reading difficulties were reported in 8 children (2.3%). 3/488 patients (0.6%) required secondary surgical intervention.

**Conclusions:**

Both the literature review and our cohort suggest that children with isolated sagittal synostosis who undergo early surgical correction generally have favorable long-term functional and neurodevelopmental outcomes.

**Supplementary Information:**

The online version contains supplementary material available at 10.1007/s00381-026-07292-y.

## Introduction

The literature is divided between studies demonstrating that surgery has positive neurodevelopmental effects and those who believe sagittal synostosis (SS) is primarily an aesthetic condition. This *specific* subject will be discussed in a different paper, within this collection (ref–Soleman et al.).

Regarding neurodevelopmental outcome in SS, the literature presents somewhat conflicting findings. For example, Boltshauser et al. [1] reported overall normal cognitive, behavioral, academic, and quality-of-life outcomes, with only mild domain-specific weaknesses that were not functionally significant [1]. However, Kalmar et al. [2] demonstrated that children with nonsyndromic single-suture craniosynostosis exhibit an increased risk of cognitive, language, attention, executive, and motor deficits, even though some early motor delays improved following surgery [2]. These opposing findings illustrate the ongoing uncertainty regarding the true neurodevelopmental impact of sagittal synostosis following surgical treatment, and the extent to which subtle deficits may be present despite generally favorable functional outcomes.

Some studies indicate that isolated SS is associated with a risk of elevated intracranial pressure (ICP), both before and after surgical intervention. Increased ICP may occur even in the absence of clear clinical symptoms, and continuous invasive monitoring has been suggested to be a sensitive tool for detecting elevated pressure without overt clinical presentation. Although early surgical correction typically results in a reduction of ICP values and resolution of papilledema when present, a small subset of patients may experience persistent or newly developing elevated ICP following surgery. Accordingly, a recent study by Sadon et al. [3] reported a very low incidence of late-onset clinical symptoms of elevated ICP and papilledema after early surgical intervention, while emphasizing the importance of long-term follow-up [3]. In addition, papilledema is considered a specific marker of elevated ICP; however, its absence does not necessarily totally exclude increased intracranial pressure, particularly in younger patients [4]. These findings highlight the complexity of diagnosing and monitoring ICP in patients with SS and underscore the importance of prolonged postoperative surveillance [4].

In this manuscript, we provide a review of the literature on the neurodevelopmental outcome in children with SS. In addition, we present our own results on *functional* outcomes among 488 children who underwent early, wide, and open strip craniectomy.

## Methods

A literature search was conducted in the PubMed and Google Scholar databases to identify studies addressing neurodevelopmental, cognitive, and *functional* outcome in children with sagittal synostosis, either following surgical treatment or managed conservatively. The search terms included combinations of: sagittal synostosis, sagittal craniosynostosis, scaphocephallus neurodevelopmental outcome, cognitive development, learning difficulties, behavioral outcome, functional outcome, quality of life, and long-term outcome. The search focused on studies that directly evaluated neurodevelopmental, cognitive functioning, academic achievement, functional symptoms, or daily adaptation. Twenty-two original research articles and six review articles (Table 1) met these criteria and reported long-term developmental or functional outcome and were, therefore, included in this review.

**Table 1:** Overview of included original studies and review articles

Article Name		First Author & Year	Patients (N)	Surgical intervention	Study design	Key Findings
Original studies:						
1	Sagittal craniosynostosis: cognitive development, behavior, and quality of life in unoperated children	Boltshauser 2003 [1]	30	Unoperated	Retrospective	Untreated isolated sagittal craniosynostosis generally shows normal cognitive development, behavior, school performance, and quality of life, with only mild domain-specific weaknesses that are not functionally significant
2	Developmental, behavioral, and cognitive outcomes in unoperated children with metopic or sagittal synostosis (CC-UK)	Qi 2023 [9]	112	Unoperated	Prospective	Unoperated sagittal synostosis generally shows normal cognitive, behavioral, and developmental outcomes, with only minor and inconsistent concerns in social and emotional domains
3	Prognosis for mental function in scaphocephaly	Arnaud 1995 [5]	396	268 operated	Prospective	Children with scaphocephaly generally show good cognitive outcomes, whether they undergo surgery or not. The strongest predictor of mental function is the initial developmental level (DQ)
4	Long-term neuropsychological effects of sagittal craniosynostosis on child development	Magge 2002 [27]	16	operated	Retrospective	Children with single-suture sagittal craniosynostosis have higher rates of learning disabilities than the general population, even after corrective surgery and despite normal IQ
5	Long-term neuropsychological development in single-suture craniosynostosis treated early	Chieffo 2010 [6]	65	Operated	Retrospective	Despite early surgical correction, children with sagittal or unicoronal craniosynostosis may show subtle long-term neuropsychological deficits in adolescence
6	Long-term neurocognitive outcomes in 204 single-suture craniosynostosis patients	Junn 2023 [28]	204	Operated	Retrospective	Surgical correction does not fully normalize neurocognitive outcomes, with metopic synostosis associated with worse long-term cognitive and visuomotor function than sagittal synostosis
7	The Cognitive Profile of Children with Nonsyndromic Craniosynostosis	Kljajić 2019 [29]	73	Operated	Retrospective	Children with nonsyndromic craniosynostosis who underwent surgery generally have average overall intelligence, but those operated on for sagittal synostosis may show subtle deficits in working memory and processing speed that could contribute to learning difficulties
8	Sustained attention and vigilance of children treated for sagittal and metopic craniosynostosis	Kljajić 2020 [30]	61	Operated	Retrospective	Children treated surgically for sagittal or metopic craniosynostosis show mild but measurable long-term deficits in sustained attention and vigilance compared with normative data, independent of surgical technique
9	Children Treated for Nonsyndromic Craniosynostosis Exhibit Average Adaptive Behavior Skills with Only Minor Shortcomings	Kljajić 2021 [31]	73	Operated	Retrospective	Children treated for nonsyndromic craniosynostosis demonstrate overall average adaptive behavior skills in daily life, with no significant differences in behavioral outcomes between surgical techniques used for sagittal synostosis
10	Long-term parental satisfaction and cosmetic, ophthalmological, and cognitive outcomes after sagittal strip craniectomy with barrel stave osteotomies and occipital release	Pinar 2025 [10]	25	Operated	Retrospective	High parental satisfaction was reported following open midline strip craniectomy with barrel stave osteotomies and occipital release for nonsyndromic sagittal craniosynostosis
11	The cognition of patients with sagittal synostosis and developmental or behavioral concerns in relation to surgical timing or technique	Kurniawan 2025 [11]	534	Operated	Retrospective	Patients with sagittal synostosis who had developmental or behavioral concerns showed slightly lower intellectual ability than the norm, and neither surgical technique nor timing affected cognitive outcomes
12	Neurocognitive sequelae of scaphocephaly	Virtanen 1999 [8]	18	Operated	Prospective	Despite early surgical correction of the skull shape, children with sagittal craniosynostosis still showed mild deficits in auditory short-term memory and language development compared with age- and gender-matched controls
13	Neurodevelopmental functioning of infants with untreated single-suture craniosynostosis during early infancy	Da Costa 2012 [32]	56	Unoperated	Prospective	Untreated single-suture craniosynostosis is associated with an increased risk of developmental delay in early infancy, particularly affecting motor skills
14	Longitudinal study of the neurodevelopmental characteristics of treated and untreated nonsyndromic craniosynostosis in infancy	Da Costa 2013 [33]	64	44 operated	Prospective	Nonsyndromic craniosynostosis is linked to developmental delays in both untreated and treated children, mainly affecting motor skills; timing of surgery does not alter outcomes
15	Multisite study of infants with single-suture craniosynostosis: preliminary report of presurgery development	Kapp-Simon 2005 [7]	100	Unoperated	Prospective	Infants with untreated single-suture craniosynostosis demonstrate lower cognitive, motor, and language scores than normative expectations, suggesting a higher developmental risk than previously assumed
16	Speech, language, and cognitive development in children with isolated sagittal synostosis	Shipster 2003 [34]	76	59 operated	Retrospective	No increased global cognitive impairment, but 37% had speech/language impairments, mostly moderate or severe; later surgery and family history increased risk
17	Deficient language acquisition in children with single suture craniosynostosis and deformational posterior plagiocephaly	Korpilahti 2012 [35]	61	49 operated	Prospective	Increased risk of early language impairment in children with single-suture craniosynostosis; 21% showed severe speech-language disorders
18	American society of maxillofacial surgeons outcome study: preoperative and postoperative neurodevelopmental findings in single-suture craniosynostosis	Cohen 2004 [36]	22	15 operated	Prospective	Mild baseline neurodevelopmental deficits in mental and motor scores; motor scores improved after surgery, but some deficits persisted at 1-year follow-up
19	Assessing Long-Term Neurodevelopment among Children with Non-Syndromic Single Suture Craniosynostosis	Kalantar-Hormozi 2022 [37]	70	Operated	Prospective	Lower verbal IQ in younger children and reduced processing speed in older children with SSC compared to healthy peers
20	Morphologic Severity of Craniosynostosis: Implications for Speech and Neurodevelopment	Tandon 2021 [38]	62	Operated	Retrospective	Morphologic severity correlates with speech-language concerns in unicoronal SSC, but not in sagittal or metopic SSC
21	Sagittal synostosis: does choice of intervention and its timing affect the long-term aesthetic and neurodevelopmental outcome? A single-institution study of 167 children	Chowdhury 2022 [39]	167	164 operated	Retrospective	Neurodevelopmental outcomes were similar regardless of type or timing of surgery
22	Neurodevelopment of infants with single-suture craniosynostosis: presurgery comparisons with case-matched controls	Speltz 2007 [40]	125	Unoperated	Prospective	Children with single-suture craniosynostosis show modest but consistent neurodevelopmental delays in cognitive and motor domains prior to surgery, independent of suture location, age, sex, or maternal IQ
Review/Meta-analysis Articles						
1	Cognitive impact of craniosynostosis	Lekovic [12]				Children with single-suture craniosynostosis are at risk of developmental delay and learning disabilities, especially affecting speech and language skills; the effect of surgery on cognitive outcomes remains unclear
2	Neurodevelopmental outcomes in infants and children with single-suture craniosynostosis: a systematic review	Knight [13]				Children with SSC are at increased risk for cognitive, language, and motor difficulties during infancy and childhood, both pre- and post-surgery
3	Neurodevelopment of children with single suture craniosynostosis: a review	Kapp-Simon [14]				SSC is associated with mild but persistent neuropsychological deficits, despite surgical correction
4	Single-suture craniosynostosis: a review of neurobehavioral research and theory	Speltz [15]				Isolated single-suture craniosynostosis is associated with a 3–fivefold increased risk of cognitive, learning, or language difficulties, with limited evidence that surgery prevents neurobehavioral impairment
5	Neurocognitive outcomes of children with non-syndromic single-suture craniosynostosis	Kalmar [2]				Children with single-suture craniosynostosis show increased risk of cognitive, language, attention, executive, and motor deficits; early motor delays improve after surgery
6	Morphological, functional and neurological outcomes of craniectomy versus cranial vault remodeling for isolated nonsyndromic synostosis of the sagittal suture: a systematic review	Thwin [16]				Children may have normal global IQ but remain at risk for specific neurodevelopmental deficits

As of our own results, we identified 507 consecutive patients diagnosed with SS who underwent early wide and open strip craniectomy surgery at the Tel Aviv Sourasky Medical Center (Ichilov Hospital), Tel Aviv, Israel, between 1999 and 2022, at an average age of 3.1 months (SD ± 0.5 months). All children were examined by a pediatrician, and none of them had significant neurological abnormalities. Of these patients, 17 did not complete the questionnaire and 2 declined participation, leaving 488 patients who met the criteria of a minimum follow-up period of two years (mean 9.4 years, SD ± 6.3, range 2–25 years) and completed a telephone questionnaire. Out of the 488 children evaluated, 346 (71%) were of school age (6 years and older), including 156 (32%) who were of high school age (12 years and older).

Data collection (see appendices A and B) included demographic information, sex, age, family history of craniosynostosis (first-degree relatives), intraoperative complications, length of hospitalization, comorbidities, mortality, postoperative follow-up, and cosmetic outcomes. All parents were asked to complete a telephone questionnaire about their experiences and their child’s development during and after the surgery.

The questionnaire and telephone conversation completed by 488 parents were designed to address functional outcome, focusing on the children’s development and schooling in the years following surgery.

When additional questions arose from the questionnaire responses, parents who reported being under neurological follow-up were contacted again by phone (*n* = 133) to obtain focused complementary clinical and developmental information. The combination of the functional questionnaire and the follow-up phone interviews allowed for a more complete and reliable assessment of the children’s f*unctional* developmental status in the years after surgery.

Appendix A represents our primary questionnaire in 488 families. Appendix B is the follow up questionnaire filled for 133 patients to further clarify functional development related issues.

Although additional data were collected from the questionnaires (e.g., cosmetic and perioperative outcomes), this manuscript focuses specifically on neurodevelopmental outcomes. Other results will be reported in a separate publication. Additionally, older patients were contacted for additional follow-up, The additional contact was made to collect responses to a supplementary questionnaire focusing on long-term outcomes, which will be analyzed in a separate manuscript.

## Results

Four hundred eighty-eight patients (397 males, 81%) out of 507 patients (96%) met the inclusion criteria and completed the telephone questionnaire. Out of all the parents who responded to the questionnaire, 82% of the questionnaires were completed by the mother (401 questionnaires). The follow-up duration ranged from 2 to 25 years (mean 9.4 years, SD ± 6.3).

Parental satisfaction with surgical outcomes was high. Overall, 95.2% of parents answered “Yes” to question B2 (“Are you satisfied with the outcome?”). In addition, 92.6% of parents reported that the child was satisfied with the result (question B1), and 99.9% answered “Yes” to question B3 (“Are you satisfied that the child underwent surgery?”). 133 patients reported being under neurological follow-up. These patients were subsequently re-contacted to complete a more detailed follow-up questionnaire. Most of these parents were referring to standard postoperative neurosurgical follow-up rather than true neurological evaluation. Only 20 of them actually had neurological problems which were classified as simple (learning disability, speech delay, and developmental delay) or serious (seizures, CP, FOXP1-related disorder, and autism spectrum disorder). which were not necessarily related to the surgery or to the craniosynostosis. (See Table 2).

**Table 2:** Neurological diagnoses among the 20 children with documented neurological problems

Neurological Diagnosis	Number of Patients (n)	Category
Learning disability	11	Simple
Speech delay	3	Simple
Developmental delay	1	Simple
Seizures	2	Serious
Cerebral palsy (CP)	1	Serious
FOXP1-related disorder	1	Serious
Autism spectrum disorder	1	Serious
Total	**20**	

Analysis of the questionnaire data showed that, overall, the vast majority of children did not exhibit reported neurodevelopmental difficulties following surgery. Among the 20 children with neurological problems, 15 had mild developmental concerns potentially related to sagittal synostosis, including learning disabilities (*n* = 11), speech delays (*n* = 3), and developmental delay (*n* = 1), while no additional academic challenges were reported for these children. (See Table 2) One patient was diagnosed with FOXP1. Two children required additional professional schooling aid, and one child needed assistance from a classroom aid in preschool. Of the children included in the study, 24 reached the age of 18 and underwent evaluation by the military recruitment office. Among these, 4 (16.7%) received an army medical profile score below 97 (the maximal score as in healthy subjects). In none of these cases was the reduction related to the surgery they had undergone. The reasons for the reduced profile army scores were as follows:

- One child with asthma
- One who was underweight
- One child who underwent an additional surgery to correct scoliosis
- One child with congenital hearing loss

3/488 (0.6%) required additional surgery. Two were for papilledema and increased ICP and 1 was to improve on an occipital hump.

## Discussion

In the reported large series described hereby, we managed to identify and receive responses of the vast majority of parents with a fair group of patients with a very long-term follow-up.

Overall, the available literature suggests that children with nonsyndromic sagittal synostosis tend to demonstrate preserved global cognitive and functional outcomes, alongside a potential risk for subtle neurodevelopmental vulnerabilities in a subset of patients. Neurodevelopmental outcomes in children with sagittal synostosis have been widely studied, with most reports suggesting preserved global cognitive and functional abilities alongside a questionable increased risk of subtle neurodevelopmental difficulties in a subset of patients. Previous studies examining developmental, cognitive, and functional outcomes have reported largely age-appropriate outcomes across both surgically treated and conservatively managed patients, with global cognitive and adaptive functioning most often described as within the normal range. Several studies, however, noted mild and selective vulnerabilities in specific neurodevelopmental domains, including attention, memory, visuospatial abilities, language, executive function, and motor skills. When identified, these vulnerabilities were typically detected through formal neuropsychological assessments and were not consistently associated with clinically significant limitations in daily functioning, academic achievement, or quality of life. The present discussion integrates these findings with our results, emphasizing overarching patterns and clinical implications rather than individual study details.

Several studies have examined developmental, cognitive, and functional outcomes in children with SS, with largely reassuring results. While not all studies focused exclusively on sagittal synostosis, we limited our analysis to sagittal synostosis-specific outcomes. We hereby selectively describe some of the more important contributions:

Arnaud et al. evaluated 396 children with scaphocephaly, including both surgically treated and conservatively managed patients, in order to examine long-term cognitive functioning and its association with surgical intervention. The results demonstrated that in the majority of cases, cognitive functioning was within the normal range, with no significant differences between the operated and non-operated groups. The cognitive evaluation was relatively global, without detailed domain-specific analysis, and the duration of follow-up was not precisely defined, limiting conclusions regarding very long-term outcome [5].

Boltshauser et al. reported on a cohort of 30 non-operated children with sagittal synostosis who were assessed after a mean follow-up of 9.25 years. The study demonstrated generally normal cognitive, behavioral, academic, and social functioning in most children; however, mild subclinical difficulties in attention and memory were identified in a subset of participants. These difficulties were detected through formal neuropsychological assessments and did not translate into impairments in everyday functioning and schooling. Nevertheless, the small sample size and the absence of a surgically treated comparison group limit the generalizability of the findings [1].

In the study by Chieffo et al. 65 children and adolescents with single-suture craniosynostosis who underwent early surgical treatment were evaluated using comprehensive neuropsychological assessments during adolescence. The findings demonstrated preserved global intellectual and adaptive functioning, alongside mild and selective cognitive weaknesses identified on formal testing, particularly in domains such as attention and visuospatial memory. These findings were not associated with significant impairments in daily functioning. However, the study did not include a healthy control group, and the clinical significance of these subtle findings remains uncertain [6].

Kapp-Simon et al. focused on cognitive functioning in infants with single-suture craniosynostosis prior to surgical intervention. The results indicated average cognitive performance within the normal range, but with a higher proportion of mild developmental delays compared with normative data. These findings suggest early developmental vulnerability in a subset of children. However, assessments conducted at a very young age limit the stability of the findings, and the study does not provide information on long-term development or postoperative outcomes [7].

The study by Virtanen et al. examined cognitive development in children with sagittal synostosis and reported generally normal global cognitive functioning, with variability across specific domains. These findings support the concept of preserved overall cognitive ability. However, the study included 18 patients, did not include a detailed neuropsychological evaluation, and domain-specific analyses were limited [8].

Da Costa et al. examined infants with untreated single-suture craniosynostosis. Developmental scores, particularly in the motor domain, were found to be slightly lower than normative values, although most children continued to function within the normal range. These findings indicate subtle early developmental delay. However, follow-up was limited to early infancy, and it remains unclear whether these differences persist later in life or are influenced by subsequent surgical intervention.

Kalmar et al. reported that children with nonsyndromic single-suture craniosynostosis exhibit an increased risk of cognitive, language, attentional, executive, and motor deficits, even following surgery, although some early motor delays improved over time. These findings present a less optimistic picture compared with other studies. However, the study population was heterogeneous and not limited to sagittal synostosis, complicating attribution of the findings specifically to this subtype [2].

In the study by Robert Qi et al. 112 non-operated children with sagittal or metopic synostosis were evaluated at ages 3 and/or 7 years. Average cognitive and developmental performance was reported, with mild and inconsistent difficulties observed in some children. These findings support generally preserved functioning with individual variability. However, assessments were limited to early childhood, and no data were provided regarding later developmental outcomes [9].

Junn et al. conducted a prospective, double-blinded cohort study of 56 children with nonsyndromic sagittal craniosynostosis, assessing neurocognitive outcomes including Full-Scale IQ and visuomotor integration. Children with damaging mutations in highly constrained genes (*n* = 18) showed significantly lower performance in these domains, independent of surgical type and age at surgery. The limited sample size and lack of functional outcome measures restrict the generalizability and clinical interpretation of these findings.

Pinar et al. reported long-term cosmetic, ophthalmological, and cognitive outcomes in 25 children with nonsyndromic sagittal craniosynostosis treated with open strip craniectomy and occipital release, with at least three years of follow-up. Using parent-reported adaptive behavior and quality-of-life measures, the study found largely age-appropriate functioning, high quality of life, and high parental satisfaction, without significant neurocognitive or ophthalmological impairment. However, the small single-center cohort, lack of a control group, and reliance on parent-reported outcomes limit the generalizability and strength of these findings [10].

Kurniawan et al. examined the cognitive profile of children with sagittal synostosis who were referred for evaluation due to developmental or behavioral concerns. In a retrospective cohort of 534 surgically treated patients, 78 children underwent psychological assessment using Wechsler intelligence scales. Mean Full-Scale IQ and Performance IQ scores were slightly lower than population norms but remained within the normal range, with more pronounced difficulties in visuospatial functioning. However, the findings were derived from a subgroup selected based on parental concern, which limits the generalizability of the results [11].

We identified six review articles that addressed single-suture craniosynostosis in general, with sagittal synostosis discussed as part of the broader context. Collectively, these studies indicate that children with single-suture craniosynostosis are at increased risk for cognitive, language, motor, and neurobehavioral difficulties. Lekovic [12] highlighted potential developmental delays and learning disabilities, particularly affecting speech and language, while noting that the effect of surgery on cognitive outcomes remains unclear [12]. Knight [13] reported that cognitive, language, and motor difficulties can occur both before and after surgery [13]. Kapp-Simon [14] described mild but persistent neuropsychological deficits despite surgical correction [14]. Speltz [15] found a 3–fivefold increased risk of cognitive, learning, or language difficulties, with limited evidence that surgery prevents neurobehavioral impairment [15]. Kalmar [2] emphasized that children with SSC show deficits in cognition, language, attention, executive function, and motor skills, though early motor delays may improve postoperatively [2]. Thwin [16] reported that while global IQ may be normal, specific neurodevelopmental deficits can persist [16]. Overall, these reviews collectively suggest that neurodevelopmental impairments are a consistent concern in single-suture craniosynostosis, and the benefits of surgery for cognitive outcomes remain partially uncertainIt is important to acknowledge that, according to multiple studies using formal neurocognitive testing, children with sagittal synostosis may exhibit a higher proportion of subtle cognitive and developmental deficits compared to the general population. While these differences are often mild and may not impact daily functioning, they cannot be excluded based on our data, as objective testing was not performed in this cohort.

Interpretation of these observations is constrained by methodological limitations reported across individual studies, including small or selected cohorts, variable and sometimes limited follow-up durations, heterogeneity in assessment tools, and the frequent inclusion of mixed single-suture craniosynostosis populations rather than sagittal synostosis alone. In addition, several studies relied primarily on global developmental or cognitive measures without detailed domain-specific analyses. Consequently, while these prior findings provide important context for the present results, uncertainty remains regarding the clinical relevance of subtle neurodevelopmental findings and the extent to which surgical intervention influences long-term outcomes. Further complicating the interpretation of these subtle findings is the fact that the normal control groups are not well defined and studied. These control groups are also heterogeneous and vary among countries, cities and communities.

Elevated intracranial pressure (ICP) has also been suggested as an additional factor influencing outcomes in children with sagittal synostosis. The issue of elevated ICP before and immediately after surgery is also somewhat confusing. Several studies have shown that children with isolated sagittal synostosis (SS) are at risk for increased intracranial pressure both prior to and immediately following surgical repair [17–20]. The reported prevalence of elevated ICP varies widely depending on patient characteristics and the methods used to assess pressure. While most patients experience a significant reduction in ICP after surgery, some may continue to exhibit persistent or recurrent elevation [17, 18, 20, 25]. Importantly, elevated ICP does not always correspond to clinical symptoms or findings such as papilledema or reduced skull growth [21].

The timing of surgical intervention may influence these ICP-related outcomes. Some evidence suggests that repair performed before six to nine months of age could be associated with lower rates of postoperative ICP elevation [22]. However, results are inconsistent across studies, likely reflecting differences in cranial morphology, suture type, surgical technique, and individual patient factors [23, 24]. Therefore, the optimal timing and reasoning for surgery to prevent ICP-related complications remain uncertain [22].

Long-term individualized follow-up is essential to monitor ICP-related issues in the years following correction, or in those not operated upon. Despite this ICP-related information, routine ICP measurement before, during, or immediately after surgery in infants with SS has largely been abandoned. Follow-up is now reserved for monitoring practical, clinically and ophthalmologically relevant changes^26^. As shown by Sadon et al., silent papilledema may develop in the years following surgery, and, although rare, it represents an important aspect of routine monitoring [3].

Our cohort demonstrates generally favorable long-term functional and parent-reported neurodevelopmental outcomes. In the context of long-term follow-up after surgical correction of sagittal synostosis, our findings indicate high levels of parental and patient satisfaction with surgical outcomes. Across the cohort, the majority of children did not demonstrate parent-reported neurodevelopmental difficulties over time. While a proportion of patients were described as being under neurological follow-up, this was most often related to routine postoperative neurosurgical surveillance rather than formal neurological assessment. Only a small subset of children was identified with neurological conditions, predominantly mild developmental concerns such as learning disabilities, speech delay, or developmental delay, without additional reported academic challenges (Table 2). More severe neurological diagnoses were rare and were not necessarily related to the surgery or to the underlying craniosynostosis. Functional outcomes in late adolescence, as reflected by military recruitment medical evaluations, remained largely preserved, and no reductions in medical profile were attributed to the surgical intervention. The requirement for additional surgical procedures was uncommon. Taken together, these findings support favorable long-term functional and developmental outcomes following surgical management of sagittal synostosis, as reflected by high satisfaction rates and a low prevalence of reported neurological morbidity. Also to mention that the incidence of such subtle challenges in the "normal" population in Israel has not been studied and reported.

### Limitations

This study is subject to inherent limitations of retrospective cohort studies. Further, as part of our routine clinical practice, normal infants do not undergo genetic testing. Similarly, *formal* neurocognitive evaluation is not routinely performed in the absence of neurological complaints or findings. Importantly, the patients did not undergo formal neuropsychological testing at the time of completing the questionnaire. Consequently, because genetic testing and formal neurocognitive evaluations were not routinely performed, some underlying genetic variants or subtle cognitive differences may not have been identified, which should be considered when interpreting the results. The aim of the current study was therefore to examine the core developmental parameters that parents typically inquire about—namely, real-life functional outcome and school performance. Accordingly, outcomes were based on parent-reported assessments, which may introduce subjectivity and reporting bias and may not include subtle neurocognitive deficits.

## Conclusions

Children with premature sagittal suture closure who undergo surgery in infancy generally demonstrate normal long-term subjective neurodevelopmental outcomes, with very low rates of mostly mild learning difficulties and rare need for educational interventions. Functional outcomes in adulthood were comparable to the general population, with no evidence of neurological or functional impairment. Although based on parent-reported assessments, these findings suggest that early surgical correction allows normal development without meaningful subjective long-term consequences.

## Supplementary Information

Below is the link to the electronic supplementary material.

ESM 1 (DOCX 19.9 KB)

## Data Availability

No datasets were generated or analyzed during the current study.
